# Ruling Out *Bacillus anthracis*

**DOI:** 10.3201/eid1004.030544

**Published:** 2004-04

**Authors:** Joseph Papaparaskevas, Dimitra P. Houhoula, Maria Papadimitriou, Georgios Saroglou, Nicholas J. Legakis, Loukia Zerva

**Affiliations:** * National and Kapodistrian University of Athens Medical School, Athens, Greece; † Hellenic Center for Infectious Diseases Control, Ministry of Health, Athens, Greece

**Keywords:** *Bacillus anthracis*, PCR, motility, hemolysis, bioterrorism, PLET agar

## Abstract

Optimization of methods for ruling out *Bacillus anthracis* leads to increased yields, faster turnaround times, and a lighter workload. We used 72 environmental non–*B. anthracis* bacilli to validate methods for ruling out *B. anthracis*. Most effective were horse blood agar, motility testing after a 2-h incubation in trypticase soy broth, and screening with a *B. anthracis*–selective agar.

The potential use of *Bacillus anthracis* as a bioterrorism agent has long been suspected. In 2001, biologic warfare became a reality (*1*), and microbiology laboratories around the world faced the problem of establishing rapid protocols for ruling out the presence of *B. anthracis* in clinical or environmental samples. To identify *B. anthracis,* both conventional and molecular methods have been applied. Presumptive identification is based on demonstrating a lack of β-hemolysis on sheep (*2*,*3*) or horse (*3*) blood agar plates and the organism’s lack of motility (*2*,*3*). *B.* *anthracis*–specific polymerase chain reaction (PCR) assays may also be applied for faster preliminary characterization of isolates. Several protocols targeting chromosomal, pXO1, or pXO2 plasmid sequences have been described (*4*–*9*).

Since September 2001, many incidents of alleged bioterrorism have occurred in Greece, as in other countries. Most samples from these incidents are being examined at our Department of Microbiology, National and Kapodistrian University of Athens Medical School. We isolated many bacilli, none of them *B. anthracis*, but encountered difficulties regarding the methods of ruling out *B. anthracis*. We realized that bacilli might produce different hemolysis patterns on blood agar plates made of blood from different species, and that the utility of the *B. anthracis-*selective agar, PLET (polymyxin, lysozyme, EDTA, thallous acetate) (*10*), was unknown for this type of isolates. We detected occasional false-positive results with PCR protocols previously evaluated by using reference strains rather than field isolates and tried to improve sensitivity of motility detection methods.

Few investigations have dealt with laboratory aspects of the recent bioterrorism attack (*9*,*11*,*12*), and a detailed evaluation of the methods for ruling out *B. anthracis* has not been reported. We used 72 environmental non–*B. anthracis* bacilli to validate and optimize methods for ruling out *B. anthracis*. We compared blood agar plates made of sheep, horse, and human blood for their ability to demonstrate β-hemolysis and three simple methods for motility detection. We also evaluated the specificity of PLET agar and four previously described PCR assays.

## The Study

During a 12-month period (10/2001–9/2002), 199 consecutive environmental specimens were submitted for possible detection of *B. anthracis.* Seventy-two *Bacillus* spp. strains were isolated; none was *B. anthracis*. Strains were stored at –70°C and retrospectively analyzed.

Hemolysis types were determined by subculturing them on 5% horse, sheep, and human blood agar plates: α-, β-, and γ-hemolysis were defined, according to standard criteria (*13*). Strong β-hemolysis was characterized as hemolysis clearly extending the colony margin, and weak β-hemolysis was characterized by a narrower hemolysis zone or slight hemolysis below colonies. Strains were additionally plated on PLET agar (*10*). Cultures were incubated at 35°C for 18–24 h in air; blood agar plates were incubated for 48 h. Motility testing was performed by using sterile H_2_O at time 0, as well as trypticase soy broth (TSB) at time 0 and after a 2-h incubation at 35°C. Part of a colony was dissolved in H_2_O and TSB and examined microscopically (*2*). Media were supplied from Bioprepare (Gerakas, Greece), except for human blood agar plates prepared in house with red blood cell units obtained from blood banks and a blood agar base (Scharlau Chemie, Barcelona, Spain).

Isolates were tested by three *B. anthracis*–specific PCR protocols amplifying a 152-, a 747-, and a 264-bp fragment of the chromosomal Ba813, the *pagA* (pXO1), and the *capC* gene (pXO2) sequences, respectively (*5*). A PCR recommended by the World Health Organization, which targets a 639-bp sequence of the chromosomal *B. anthracis* S-layer gene, was also performed (*8*). Crude DNA was extracted by boiling colonies in H_2_O. Control strains included the NC08234-03 *B. anthracis* Sterne strain (pXO1^+^/pXO2^−^), a *B. anthracis* strain isolated from the malignant pustule of an agricultural anthrax patient, and the *Bacillus subtilis* EO-1 reference strain (kindly provided by the Hellenic Agricultural Ministry). Two microbiologists independently assessed all results. The Fisher exact test was performed for the statistical analysis.

Diverse hemolytic activity was demonstrated by the 72 bacilli on different blood agar plates. At 24 h, strong β-hemolysis was produced by 55 (76%), 41 (57%), and 55 (76%) strains on human, sheep, and horse blood agar plates, respectively, while 6 (8%), 10 (14%) and 7 (10%) strains demonstrated weak β-hemolysis (Table). Prolongation of incubation to 48 h resulted in increased detection of strong β-hemolysis on all media.

**Table:** Numbers (%) of environmental *Bacillus* spp. isolates exhibiting various types of hemolysis on human, sheep, and horse blood agar plates at 24 and 48 hours of incubation

	Incubation time											
	24 h						48 h					
Type of hemolysis	Human^a^		Sheep^a^		Horse^a^		Human		Sheep		Horse	
	No	(%)	No	(%)	No	(%)	No	(%)	No	(%)	No	(%)
α	1	(1.4)	5	(6.9)	0	(0)	0	(0)	0	(0)	1	(1.4)
γ	10	(13.9)	16	(22.3)	10	(13.9)	4	(5.6)	9	(12.5)	2	(2.8)
Total β	61	(84.7)	51	(70.8)	62	(86.1)	68	(94.4)	63	(87.5)	69	(95.8)
Strong β	55	(76.4)	41	(56.9)	55	(76.4)	65	(90.3)	55	(76.4)	66	(91.6)
Weak β	6	(8.3)	10	(13.9)	7	(9.7)	3	(4.1)	8	(11.1)	3	(4.2)
Total	72		72		72		72		72		72	

^a^Human, sheep and horse refer to the respective blood agars.

Both *B. anthracis* strains produced γ-hemolysis on all media at 24 h, except for slight β-hemolysis below areas of confluent growth on human blood agar plates. After 48 h they remained γ-hemolytic on horse and sheep blood agar plates; however, both produced strong β-hemolysis on human blood agar plates.

At time zero of incubation, motility was detected in 33 (46%) and 45 strains (63%) examined in H_2_O and TSB, respectively. All isolates motile in H_2_O were also motile in TSB. The 2-h incubation step in TSB detected another 13 motile strains (total number of motile strains, 58; 81%) and made recognition of motility much easier.

The *capC* and *pagA* gene sequences were not amplified; however, the Ba813 sequence was amplified in seven strains (10%; specificity 90%), and the S-layer sequence was amplified in another two strains (3%; specificity 97%). Twenty-four isolates (33%) grew on PLET; 21 of them were uniformly β-hemolytic. A positive correlation between the ability to grow on PLET and BA813 PCR-positivity was detected. Five out of 24 strains (21%) that grew on PLET were positive by this PCR in comparison with 2 of 48 strains (4%) that did not grow on PLET (Fisher exact test, p = 0.037).

## Conclusions

Although other genera are known to produce distinct hemolysis types on different blood agar plates (*14*), comparative studies for bacilli have not been reported. Most environmental bacilli in our study were β-hemolytic. However, various blood agar plates manifested different abilities to support the expression of β-hemolysis as well as to demonstrate weak and strong β-hemolysis. Weak β-hemolysis must thus be interpreted with caution; isolates will be incubated for another 24 h or considered nonhemolytic strains. β-hemolysis was easier to recognize on all media after 48 h, but sheep blood agar plates were the least effective medium in detecting β-hemolysis. Finally, β-hemolysis results obtained with horse and human blood agar plates, although not identical, were usually in agreement and differed from those obtained with sheep blood agar plates (data not shown).

The production of strong β-hemolysis on human blood agar plates by the *B. anthracis* strains was unexpected, as this organism has been considered traditionally nonhemolytic. Recently, however, the ability of *B. anthracis* to express β-hemolysis was reported (*15*,*16*). Broth culture supernatants possessed hemolytic activity against human and sheep erythrocytes, whereas richness of media affected hemolysis expression (*15*). Another study demonstrated the induction of strong β-hemolysis on human but not sheep blood agar plates under anaerobic conditions (*16*). In fact, a study conducted in 1957 reported 45 β-hemolytic strains among 120 *B. anthracis* isolates that had been cultured on rabbit blood agar plates (*17*). Therefore, withholding the use of human blood agar plates would be prudent; horse blood agar plates should be used as the most “informative” medium.

According to our findings, the 2-h incubation of bacilli in TSB greatly improves recognition of motile strains. Increased motility detection using TSB rather than H_2_O has been demonstrated with 12 non–*B. anthracis* strains (*18*), though test performance after incubation was not assessed. Apparently, motile bacilli become immobilized in distilled water, while the practice of incubating them in a rich broth until they reach exponential growth phase was actually recommended in the past (*19*).

All bacilli tested negative for the presence of *capC* and *pagA* sequences, but seven strains were positive for Ba813. All were strongly β-hemolytic and five were motile. These results prompted us to assess the specificity of the S-layer PCR, which, to our knowledge, has not been evaluated before. Only two strains, different from the above seven, were positive: a motile, strongly β-hemolytic strain and a nonmotile, γ-hemolytic strain.

With the exception of the Laboratory Response Network real-time PCR (*9*), the specificity of other PCR protocols (*4*–*8*,*20*) has not been evaluated before by testing field isolates from suspected bioterrorism incidences. False-positive results have been reported with other *cap* sequence PCR assays (*4*,*6*), although efforts to establish a specific chromosomal assay have been frustrating (*6*,*7*,*20*). As *B. anthracis* strains cured from one or both plasmids exist naturally or may be obtained in vitro (*3*), and false-negative results may be encountered with plasmid-specific PCR assays (*4*), the availability of a chromosomal PCR is desirable. Our results indicate that to preserve the positive predictive value of the evaluated molecular tests, chromosomal assays should always be performed in conjunction with plasmid PCR.

Because environmental and, to a lesser extent, clinical samples may be heavily contaminated, a selective medium for *B. anthracis* would be useful. PLET is used in environmental investigations of agricultural anthrax outbreaks (*3*), because it inhibits other bacilli and gram-negative rods (*10*). In our study, the specificity of PLET was low, but PLET is still valuable, because by inhibiting two thirds of contaminating bacilli as well as other bacteria, background will decrease and isolating colonies will be easier and faster. However, the characteristic colony morphology of the two *B. anthracis* strains on blood agar was not reproduced on PLET. Examination of a large number of *B. anthracis* strains is required to confirm these observations.

A positive correlation was detected between the ability to grow on PLET and Ba813 PCR-positivity. Of note, non–*B. anthracis* strains previously reported to be Ba813 PCR–positive were isolated by using PLET (*20*). Ba813-positive bacilli may be very closely related to *B. anthracis* and demonstrate, therefore, phenotypic similarities like the ability to grow on PLET.

In conclusion, horse blood agar plates provide better recognition of β-hemolysis, and testing after a 2-h-incubation in TSB improves motility detection. The application of these tests along with PLET agar will have a substantial impact on public health laboratories that process large numbers of specimens. Workload will decrease, and the presence of *B. anthracis* will be ruled out faster, leading to earlier termination of chemoprophylaxis and diminished anxiety of exposed persons. Standardization and validation of molecular assays as direct detection tests will further decrease turnaround time; however, these methods only complement conventional testing. Selective or differential media and further refinement of conventional techniques will still be needed.
